# Research on Properties and Microstructure of Waste Polyurethane/Crumb Rubber Composite Modified Asphalt

**DOI:** 10.3390/ma19183945

**Published:** 2026-09-17

**Authors:** Zhaoyan Ye, Lu Huo, Bei Chen, Yingchun Cai, Junjie Li, Zhicong Liu

**Affiliations:** 1 School of Management, Zhengzhou University, Zhengzhou 450001, China; yezhaoyan@126.com; 2 School of Water Conservancy and Transportation, Zhengzhou University, Zhengzhou 450001, China; 3 School of Intelligent Perception and Instrumentation, Zhongyuan University of Technology, Zhongyuan Road No. 41 of Zhengzhou, Zhengzhou 450007, China

**Keywords:** waste polyurethane, crumb rubber, composite modified asphalt, viscoelastic behaviors, microcharacteristics

## Abstract

The disposal of waste polyurethane (WPU) poses a significant environmental challenge. This study investigates the use of WPU and crumb rubber (CR) as composite modifiers for asphalt, aiming to enhance binder high-temperature rheological behavior while offering an alternative recycling route for solid polymer waste. Formulated via high-shear blending across various modifier dosages, the WPU/CR composite asphalts underwent a comprehensive evaluation protocol. The binder’s performance was systematically evaluated using conventional physical tests, rotational viscosity, temperature sweep, and multiple stress creep recovery (MSCR) tests. Microstructural and chemical mechanisms were analyzed via fluorescence microscopy (FM) and Fourier-transform infrared spectroscopy (FTIR). The results demonstrate that integrating WPU into crumb rubber-modified asphalt (CRMA) yields remarkable improvements in both high-temperature rheological stability and resistance to permanent deformation. This structural upgrade is driven by a two-fold synergistic effect: the physical swelling of the CR and WPU modifiers via the absorption of light asphalt fractions, coupled with the physical intertwining, particle reinforcement, and polymer network formation within the binder matrix. However, the microstructural analysis revealed a critical trade-off: excessive WPU content led to particle agglomeration and phase separation. WPU/CR composite-modified asphalt is a promising material, but the modifier ratio is critical for balanced performance. To achieve balanced overall performance, the study recommends a composite formulation combining the selected 15% CR reference matrix with 15% WPU. Ultimately, this work delivers a viable technical framework for the premium recycling of WPU within highway engineering.

## 1. Introduction

Polyurethane is a high-performance organic polymer widely employed across construction, automotive, medical, and consumer product sectors [1,2]. According to research data, global annual polyurethane production is projected to reach 31 million tons by 2030, which will inevitably increase the generation of polyurethane waste [3]. However, the current disposal methods for WPU, mainly landfilling and incineration, bring notable environmental risks. Landfilling can lead to soil compaction and the leaching of heavy metal ions. Meanwhile, pyrolysis and incineration often release persistent organic pollutants, such as dioxin-like compounds [4]. Driven by the macro-strategies of carbon peaking and neutrality, advancing the technological upcycling of waste polyurethane represents a critical imperative. Such innovative valorization not only mitigates plastic pollution, but also acts as a fundamental catalyst for transitioning toward a resource-efficient society.

Conventional asphalt matrices are notoriously prone to permanent deformation at elevated temperatures and fracture under cold conditions. Although single modifiers such as CR and WPU have been introduced to improve these properties, their effects remain incompletely understood [5,6,7]. For example, Tahami et al. [8] used a dry-process CR modification method to increase CR content and improve high-temperature performance. However, the resulting asphalt mixture showed weak resistance to water damage and lower strength compared to the control group. Similarly, Radeef et al. [9] reported that CRMA experienced significant performance degradation under moisture exposure and long-term aging. To address these issues, viscosity-enhancing agents or wet-process techniques were required to improve durability. In a similar vein, research by Li, Zhuang, et al. [10,11] corroborates that while PU modification excellently fortifies asphalt against UV aging, thermal-oxidative aging, and high-temperature instability, its impact on cold-weather performance is marginal at best, often exacerbating low-temperature embrittlement. In 2014, to address the performance limitations of these two materials as individual modifiers, Yao et al. [12] proposed a composite modification approach to achieve synergistic enhancement.

Against this background, researchers have focused on composite modification strategies coupling CR with polyurethane. Polyurethane effectively mitigates the high-temperature deficiencies of CRMA, whereas CR counters the low-temperature brittleness typical of polyurethane-modified systems. This complementary synergy not only delivers well-balanced improvements in thermal stability, crack resistance, and fatigue life, but also sustains cost-efficiency, highlighting the superior efficacy of dual-modifier systems. For instance, Gao et al. [13] demonstrated that incorporating waterborne polyurethane and CR in an equimass ratio (1:1) substantially reinforced the binder’s viscoelastic performance across wide temperature regimes. Concurrently, the corresponding asphalt mixture exhibited enhanced resistance to permanent deformation and moisture-induced damage, alongside a 17.5% reduction in overall road construction expenditure through optimized processing. Furthermore, Zhu et al. [14] developed a composite binder comprising 8% thermoplastic polyurethane and 10% CR that established a 3D interpenetrating polymer network; rheological characterizations confirmed elevated elastic recovery and low-temperature creep rates, while molecular dynamics simulations corroborated interphase thermodynamic compatibility. These insights establish a multi-scale theoretical and empirical framework for engineering eco-friendly, durable pavement materials. Similarly, multi-scale microstructural evolution studies have proven crucial in understanding other polymer-modified cementitious composites [15].

**Figure 1:**
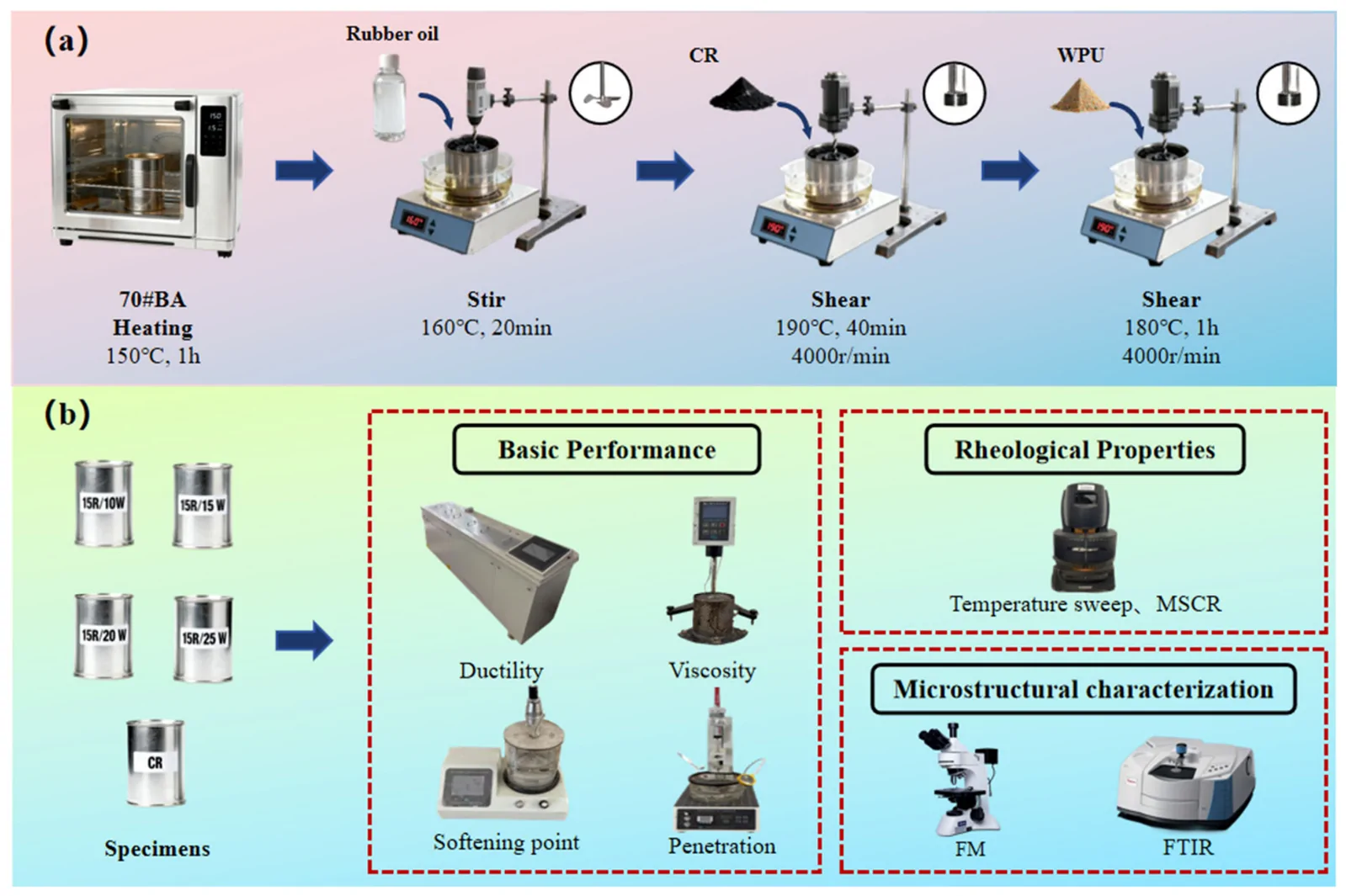
Flowchartof experimental planning. (**a**) Preparation; (**b**) Testing.

Despite preliminary efforts on polyurethane/rubber combinations, a significant research gap remains regarding the use of mechanically pulverized waste polyurethane at higher dosage levels in composite asphalt binders. Specifically, the trade-off between rheological rutting resistance and construction workability, the microstructural dispersion morphology, and the interfacial interaction mechanisms under different WPU dosages are not fully elucidated. Therefore, this study systematically evaluates the technical performance, viscoelastic rheological properties, and microscopic features (via FM and FTIR) of WPU/CR composite-modified asphalt binders. The findings aim to elucidate the dosage-dependent performance evolution and provide technical reference data for utilizing waste polyurethane in asphalt binder modification. Initially, empirical physical tests and rotational viscometry were performed to establish the baseline characteristics of the composite binders. To further appraise high-temperature rutting resistance and elastic restorative capacity, dynamic rheological measurements and MSCR tests were conducted systematically. The overarching experimental framework and research workflow are depicted in Figure 1, comprising the binder fabrication protocol in Figure 1a and the comprehensive testing methodology in Figure 1b.

## 2. Materials and Methods

### 2.1. Materials

#### 2.1.1. Base Asphalt

Table 1 summarizes the fundamental physical and technical indices of the base binder—a commercial SK-70 petroleum asphalt (60/80 penetration grade, SsangYong, Seoul, Republic of Korea)—characterized in compliance with JTG E20-2011 standard test methods [16]. This virgin asphalt was utilized as the primary substrate for preparing both neat CRMA and WPU/CR composite-modified binders.

#### 2.1.2. Crumb Rubber

The CR modifier employed in this research was supplied by Hebei Zengli Rubber Technology Co., Ltd. (Dingzhou, China), which reprocesses discarded elastomeric items including automotive tires, footwear soles, and industrial sealing rings. Through an ambient-temperature mechanical pulverization route comprising sequential cutting, extrusion, and milling, the vulcanized rubber blocks were converted into homogeneous 80-mesh particulates (particle-size limit <0.18 mm, referring to the aperture of the 80-mesh sieve). Table 2 details the primary physicochemical properties and technical specifications of the resulting CR.

#### 2.1.3. Waste Polyurethane

**Figure 2:**
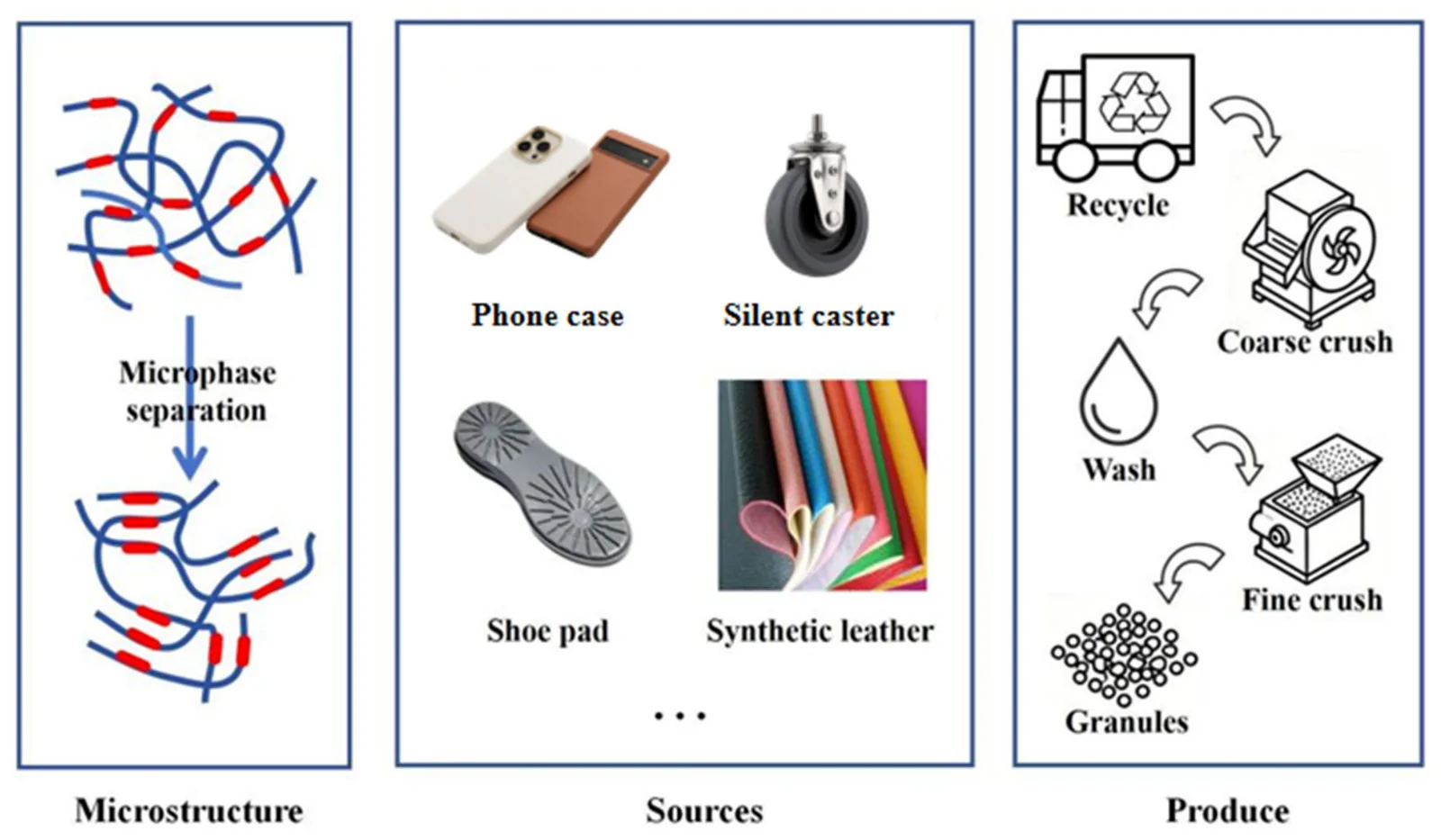
Structure, source, and synthesis of WPU.

The recycled waste polyurethane (WPU) is sourced from Lianyungang Hongrun Renewable Resources Co., Ltd. (Lianyungang, China). Compositionally, it is identified as a polyether-based thermoplastic polyurethane featuring alternating polyether soft segments and diisocyanate rigid domains (Figure 2), with cellular pores formed via physical foaming during original molding rather than thermoset covalent cross-linking. Prior to blending, the washed and dried scraps (<0.4 wt% moisture) were mechanically pulverized and screened through a 200-mesh sieve; the particle-size limit (<0.075 mm) refers to the aperture of the 200-mesh sieve, and the median diameter (D50 ≈ 42.8 μm) was determined by laser-diffraction particle-size analysis of the pulverized WPU powder. The processed WPU exhibits a density of 1.16 g/cm^3^, a tensile strength of 42 MPa, a tear strength of 64 N/mm, and a distinct softening point of 71 °C. Thermogravimetric analysis (TGA, 10 °C/min under N_2_) confirmed an initial thermal decomposition temperature (T_5%_) of 232 °C, ensuring thermal durability without polymer breakdown during high-shear asphalt mixing at 180 °C [17,18,19,20].

### 2.2. Preparation of Modified Asphalt

All modified asphalt samples in this study were prepared using a FLUKO FM300-Digital high-shear emulsifier (Chuangyi Limited Company, Shanghai, China). Throughout the experiments, the additive contents are uniformly expressed as mass percentages relative to the base asphalt.

#### 2.2.1. Preparation of CRMA

First, the base asphalt was preheated to 160 °C and blended with 3 wt% rubber oil for 20 min. Subsequently, the system temperature was raised to 190 °C, and different proportions of CR (10%, 15%, 20%, and 25% by weight) were incorporated. After continuous shear mixing at 4000 rpm for 40 min, the resulting crumb rubber-modified asphalt samples were labeled as 10 R, 15 R, 20 R, and 25 R, respectively.

#### 2.2.2. Preparation of WPU/CR Composite Modified Asphalt

Using the CRMA prepared at the selected reference CR dosage (15 wt%) from Section 2.2.1 as the baseline matrix system, the temperature was maintained at 180 °C, and various proportions of WPU particles (0%, 10%, 15%, 20%, and 25%) were added step by step. Afterwards, high-shear blending was continued at 4000 rpm for 1 h to obtain the final WPU/CR composite-modified asphalt.

## 3. Test Procedures

To ensure statistical reliability, all empirical indices—including penetration, softening point, ductility, and rotational viscosity—were evaluated across three parallel replicates (n = 3). Meanwhile, dynamic rheological measurements (temperature sweep and MSCR protocols) were performed in duplicate (n = 2) to verify experimental repeatability.

### 3.1. Test Scheme for the Reference CR Dosage

With the objective of establishing a representative crumb rubber dosage for composite modification, neat bitumen was blended with varying CR weight percentages (10%, 15%, 20%, and 25%). The prepared CRMA formulations were subjected to routine empirical evaluations, specifically 25 °C penetration, softening point, and 5 °C ductility. Based on the empirical balance between high-temperature stiffness and low-temperature flexibility, alongside workability considerations for subsequent high-dosage polymer blending, a selected reference dosage was identified to serve as the baseline matrix for WPU composite modification.

### 3.2. Basic Performance Tests

#### 3.2.1. Basic Performance Tests of Modified Asphalt

**Figure 3:**
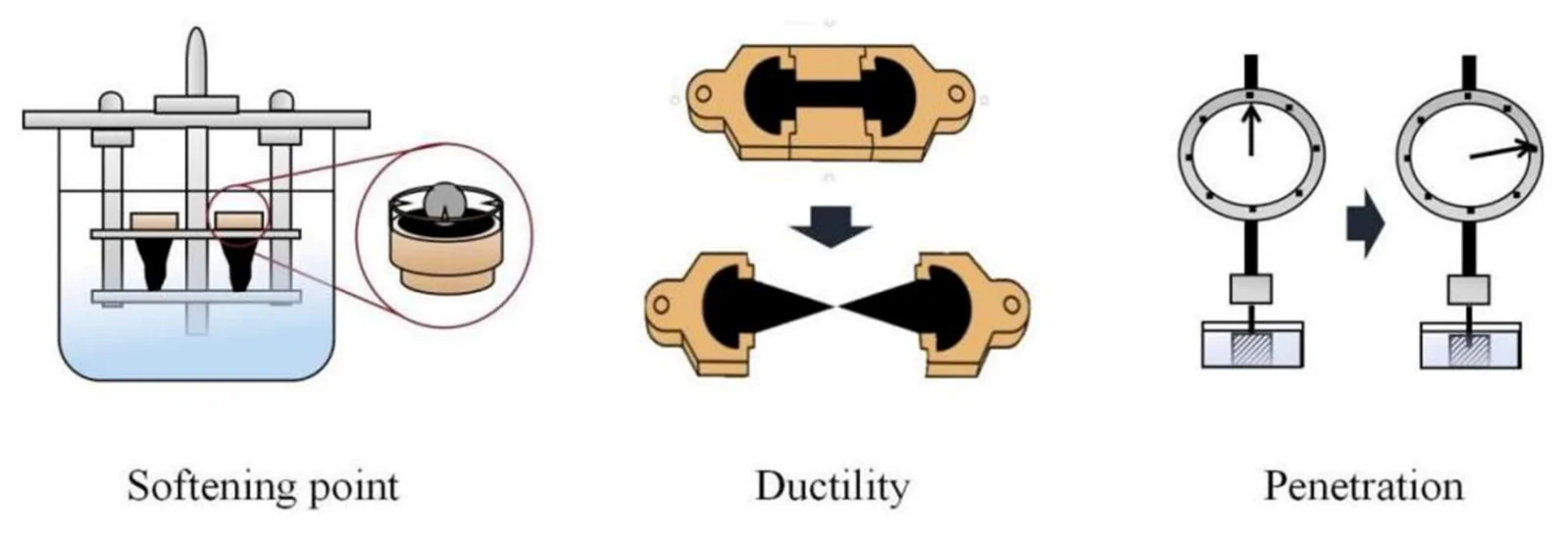
Schematic diagram of the three conventional performance tests for asphalt.

The conventional properties of WPU/CR composite-modified asphalt binders with varying WPU dosages were evaluated in accordance with the JTG E20-2011 standard test methods [16]. The key indicators of the three conventional performance tests consisted of penetration (25 °C), softening point, and ductility (5 °C), as illustrated in Figure 3. Additionally, the penetration index (*PI*) was calculated to evaluate temperature susceptibility. A higher PI indicates improved thermal stability. The PI was derived using Equation (1) [21]

(1) $$PI=\frac{1952-500\mathrm{lg}(P25)-20Sp}{50\mathrm{lg}(P25)-Sp-120}$$

where *Sp* is the softening point and P25 is the penetration at 25 °C.

#### 3.2.2. Rotational Viscosity Tests of Modified Asphalt

Viscosity is critical for assessing asphalt workability during construction. The Brookfield rotational viscosity test minimizes operator bias and effectively captures the influence of WPU and CR on binder flow behavior. Using an NDJ-1C viscometer (Changji, Shanghai, China), viscosity was measured across five temperature intervals (135 °C to 175 °C, in 10 °C steps). A temperature–viscosity response curve was constructed to illustrate the material’s thermal sensitivity.

### 3.3. Rheological Property Tests

#### 3.3.1. Temperature Sweep Tests

**Figure 4:**
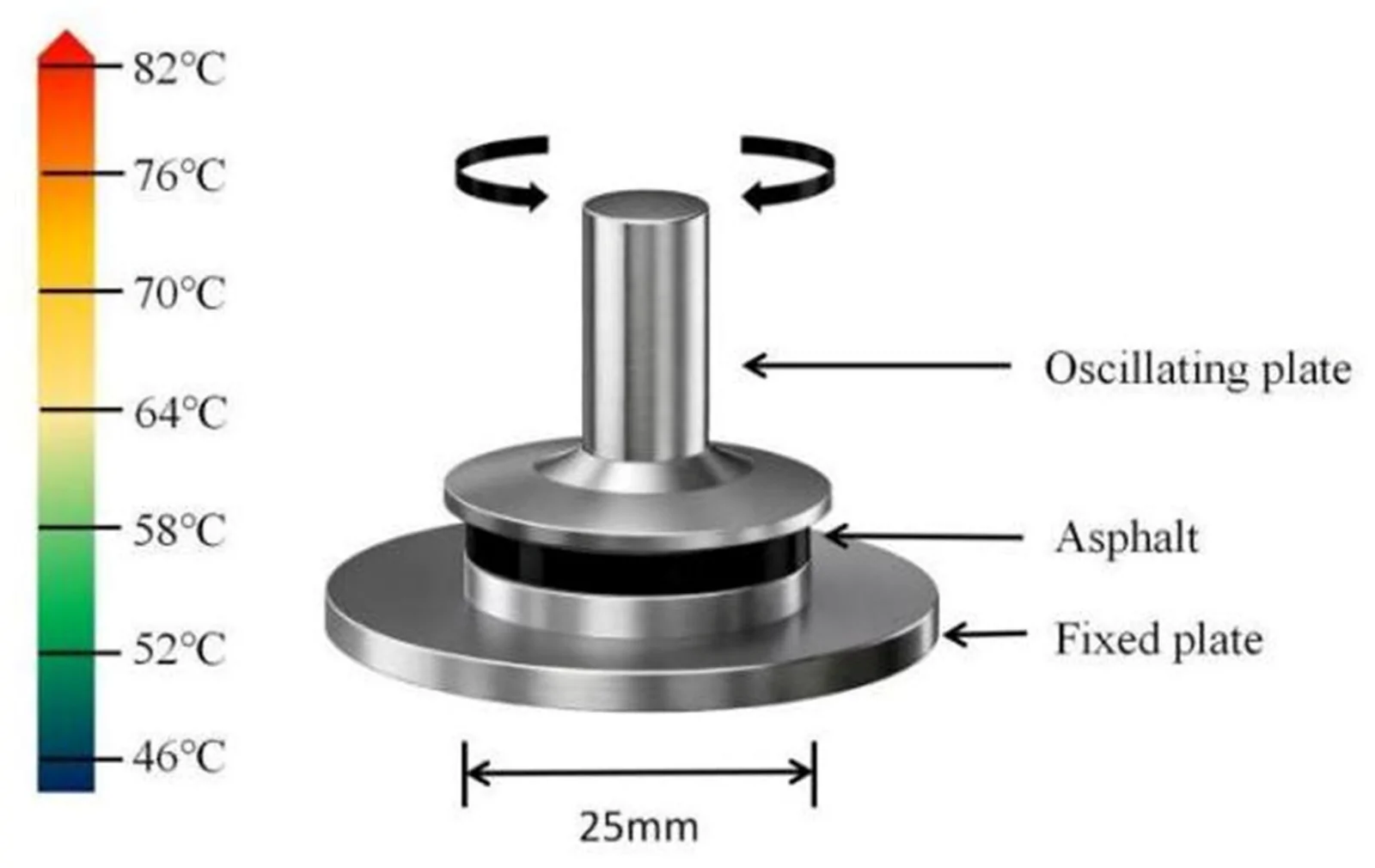
Schematic diagram of the temperature sweep test.

Dynamic temperature sweep analyses were executed in accordance with ASTM D7175 [22]. A TA-AR 1500 EX dynamic shear rheometer (Zhuokang Trading Co., Ltd., Shanghai, China) configured with 25 mm parallel plates and a 1 mm gap clearance was utilized for the characterization. Operating under a strain-controlled mode, the oscillatory shearing was applied at a constant angular frequency of 10 rad/s and a strain target of 12%. As depicted in Figure 4, the thermal evaluation spanned seven distinct temperature stages ranging from 46 °C to 82 °C at 6 °C intervals. Throughout this process, key viscoelastic indices—specifically the complex shear modulus (G*), phase angle (*δ*), storage modulus (*G*′), loss modulus (*G*″), and rutting parameter (G*/*sinδ*)—were measured and documented.

#### 3.3.2. Multiple Stress Creep Recovery (MSCR) Tests

The MSCR characterization served to evaluate the resistance to rutting of the binders under stress fields representative of traffic loading [23,24]. Following rolling thin-film oven conditioning per AASHTO T240 [25] to simulate short-term aging, the aged residues were characterized on a TA-AR 1500 EX dynamic shear rheometer across four temperatures (58, 64, 70, and 76 °C) in accordance with AASHTO T350 [26]. Prior to data recording, 10 creep-recovery conditioning cycles at 0.1 kPa were conducted to eliminate the initial loading history. Subsequently, two shear stress levels, 0.1 kPa and 3.2 kPa, were successively imposed to evaluate binder performance in linear viscoelastic and nonlinear regimes. Each stress stage comprised 10 successive 10 s cycles, each consisting of a 1 s creep phase followed by a 9 s unrestricted recovery phase. Based on the transient deformation recovery records, key high-temperature performance indicators—namely, percent recovery (*R*), non-recoverable compliance (*J_nr_*), and stress sensitivity (*J_nr-diff_*)—were calculated for binders blended with varying WPU fractions using the expressions below:

(2) $$J_{nr}=\frac{\varepsilon_{r}-\varepsilon_{0}}{\delta }$$

(3) $$J_{nr-diff}=\frac{J_{nr(3.2\mathrm{kPa})}-J_{nr(0.1\mathrm{kPa})}}{J_{nr(0.1\mathrm{kPa})}}$$

where $\varepsilon_{0}$ is the initial deformation and $\varepsilon_{r}$ is the residual deformation after 9 s of recovery; *τ* is the shear stress; *J_nr_* is the non-recoverable creep compliance; and *J_nr-diff_* is the stress sensitivity index of the non-recoverable creep compliance. In this study, *J_nr-diff_* is reported as a dimensionless ratio.

### 3.4. Microstructure Analysis

#### 3.4.1. Fluorescence Microscope Tests

To visualize the morphological state and dispersion of WPU and CR within the asphalt matrix, FM imaging was performed using an Opal fluorescence microscope (Toylezhi Technology Co., Ltd., Shanghai, China). To ensure reliable comparability across all formulations, all modified asphalt binders were prepared under the standardized shear duration (180 °C, 4000 r/min for 1 h) to reach a stabilized state. Subsequently, a small quantity of the prepared binder was deposited onto a glass slide and covered with a coverslip. The specimen was then maintained at 130 °C for 20 min to produce a relatively uniform thin layer. Following cooling to ambient temperature, FM images were collected to examine the morphology and dispersion characteristics of the incorporated modifiers within the asphalt matrix.

#### 3.4.2. Fourier Transform Infrared Spectroscopy (FTIR) Test

ATR-FTIR spectroscopy was employed to examine the chemical interactions and interfacial compatibility between the asphalt binder and the incorporated modifiers. Spectral measurements were conducted over a wavenumber range of 4000–525 cm^−1^, enabling the identification and monitoring of characteristic functional groups and organic components [27]. The obtained spectra were further analyzed to clarify the possible chemical interactions and reaction mechanisms between asphalt and WPU. In particular, the analysis was used to assess the influence of different WPU contents on the molecular structure and chemical environment of the asphalt binder.

## 4. Results and Discussion

### 4.1. Evaluation and Selection of the Reference CR Dosage

**Figure 5:**
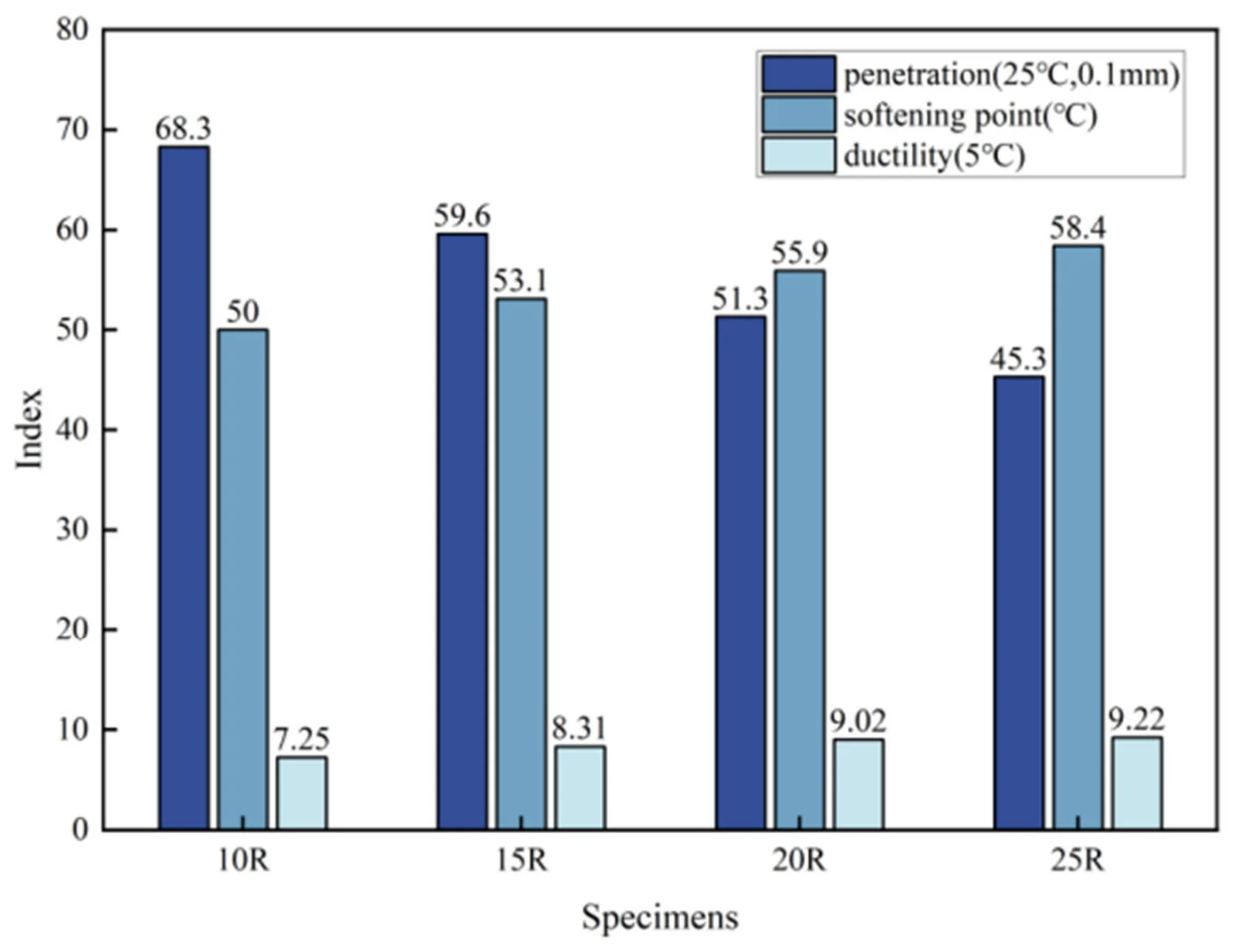
Effect of CR content on the fundamental properties of modified asphalt.

As shown in Figure 5, compared with the base asphalt, the penetration of the modified asphalt gradually decreased with the addition of CR, while the softening point and ductility continuously increased, indicating enhanced binder stiffness and improved low-temperature tensile ductility. Although 20% and 25% CR yielded higher conventional stiffness and ductility, earlier studies indicate that crumb rubber-modified asphalts exceeding 15% dosage exhibit elevated initial viscosity and significant high-shear processing resistance. Because the planned composite modification involves introducing high dosages (up to 25%) of solid WPU particles—which further increases solid volume filling and viscosity—excessive initial binder viscosity would severely hinder the subsequent incorporation and uniform dispersion of WPU. Therefore, 15% CR was selected as the baseline reference dosage (15 R) to achieve a balanced initial binder state capable of accommodating substantial WPU addition while maintaining laboratory shear workability. Based on the varying contents of WPU incorporated subsequently, the WPU/CR composite-modified asphalt samples prepared with mass fractions of 0%, 10%, 15%, 20%, and 25% WPU were designated as 15 R/0 W (or 15 R), 15 R/10 W, 15 R/15 W, 15 R/20 W, and 15 R/25 W, respectively.

### 4.2. Basic Performance

#### 4.2.1. Basic Performance of Asphalts

**Figure 6:**
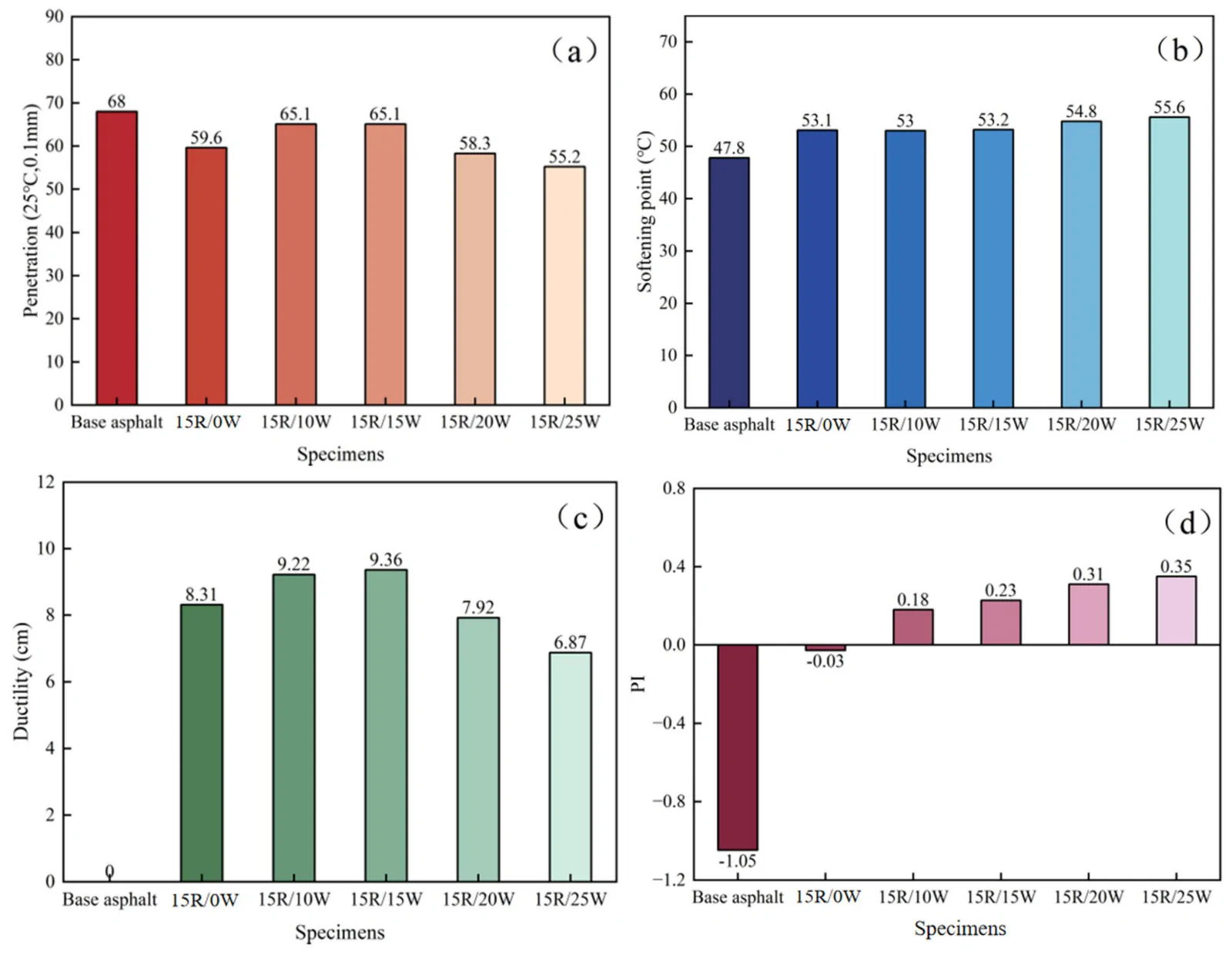
Effect of WPU content on the conventional properties of modified asphalt. (**a**) Penetration, (**b**) softening point, (**c**) ductility, (**d**) penetration index (PI).

The effects of WPU incorporation levels on the empirical physical performance of the modified asphalt are depicted in Figure 6. Introducing 15% CR remarkably enhances the matrix asphalt performance, evidenced by a drop in penetration alongside increases in both softening point and 5 °C ductility, validating the dual efficacy of CR in reinforcing thermal stability and low-temperature tensile deformability. Within the composite systems, incorporating a minor fraction of WPU initially promotes both penetration and ductility; nevertheless, elevating the modifier content further triggers a steady decline in both metrics, accompanied by a moderate rise in the softening point. Compared with the 15% CRMA control, blending 25% WPU leads to a 7.4% reduction in penetration and a 17.3% loss in 5 °C ductility, whereas the softening point remains essentially stable. The inclusion of WPU produces a notable improvement in the high-temperature stiffness of the composite-modified binder, but the concurrently reduced ductility at 5 °C suggests an impairment of its low-temperature tensile response. In summary, the empirical physical characterization demonstrates that WPU exerts a distinct stiffening effect on the WPU/CR composite binder, which provides better high-temperature performance at the expense of low-temperature flexibility.

The thermal susceptibility results illustrated in Figure 6d reveal that both CRMA and WPU/CR composite-modified binders achieve substantially higher penetration index (PI) values than the neat asphalt baseline. A progressive upward shift in PI is observed with increasing WPU dosages, corroborating the synergistic role of CR and WPU in mitigating binder temperature sensitivity. Consequently, WPU/CR composite formulations display superior thermal stability compared to single CRMA. This enhanced resistance to thermal fluctuation originates from the stable sol-gel skeleton and resilient restorative capacity established within the WPU/CR composite matrix.

The underlying mechanism is dual-faceted. Firstly, the added CR and WPU absorb light components, such as aromatic and saturate fractions, in the asphalt. This physical swelling process increases the volume fraction of the polymer phase and enriches the residual asphalt with heavier asphaltene components, thereby increasing its hardness and consistency. In addition, the physical interlocking and chain entanglement formed between the softened WPU domains and the swollen CR particles establish a cohesive three-dimensional polymer network. This interconnected skeleton constrains the translational mobility of asphalt constituents, thereby augmenting the isothermal shear strength of the binder, which translates macroscopically into enhanced stiffness and consistency [21,28]. Elevating the WPU content further consolidates this skeletal architecture. The intensified steric hindrance among polymer chains effectively mitigates the kinetic motion of asphaltene clusters exposed to thermal excitation, substantially upgrading the thermal stability of the composite system. Nevertheless, excessive WPU loading inherently stiffens the binder, reducing its low-temperature compliance and tensile deformability, which eventually impairs its low-temperature tensile deformation behavior.

#### 4.2.2. Brookfield Rotational Viscosity Tests

**Figure 7:**
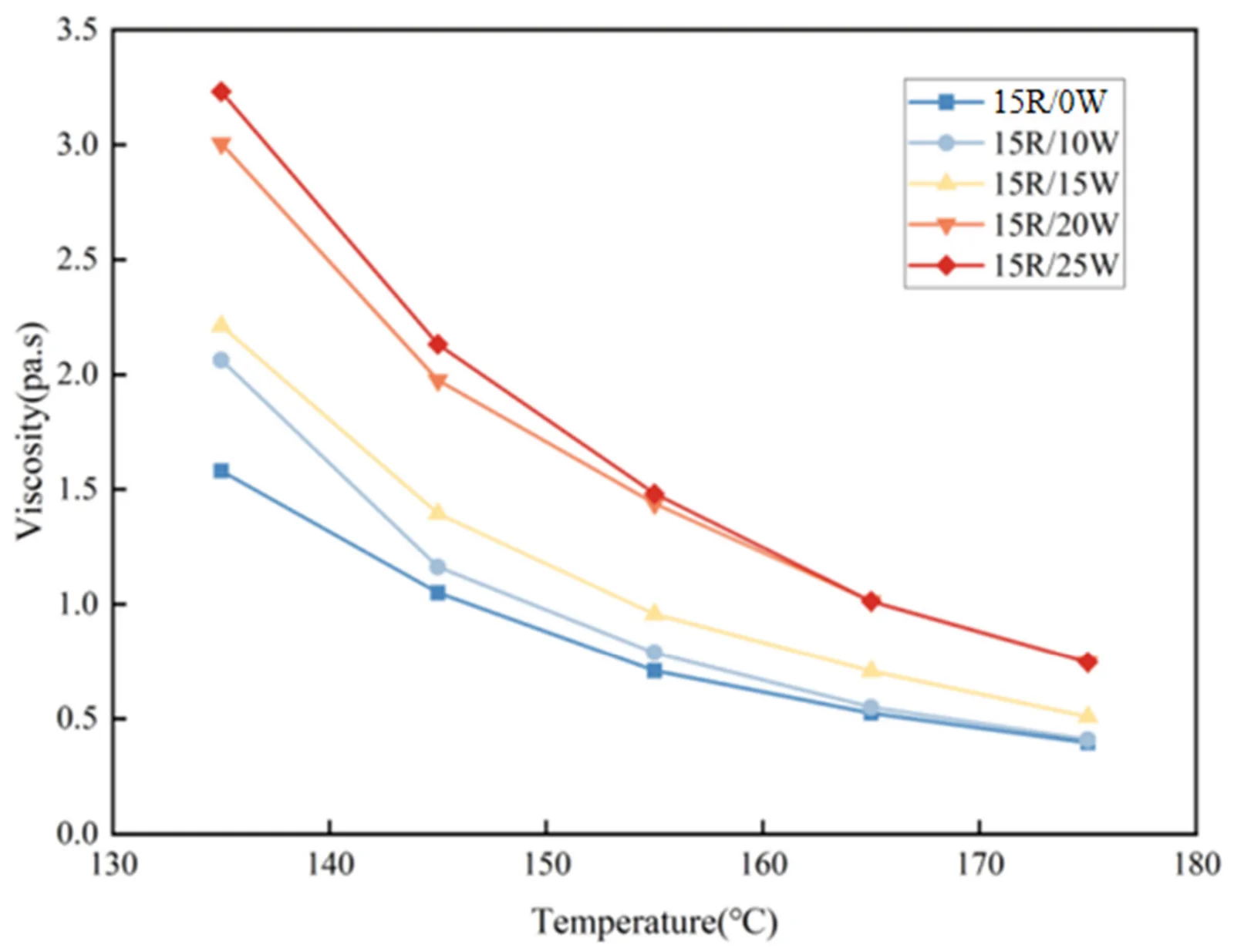
Brookfield Viscosity of Composite Modified Asphalt with Varying Contents.

The rheological response of WPU/CR composite binders under varying thermal and dosage conditions is outlined in Figure 7, where viscosity exhibits an inverse relationship with temperature and a positive correlation with WPU loading. This thickening behavior stems from the physical swelling of WPU and CR together with the mutual interlocking of the softened polymer domains, which jointly construct a robust three-dimensional polymer skeleton. Higher WPU additions intensify the physical entanglement and filler packing density within this spatial network, generating greater hydrodynamic drag on the viscometer spindle. According to both JTG F40-2004 and SHRP specifications, the maximum allowable rotational viscosity for polymer-modified asphalt at 135 °C is set at 3.0 Pa·s to ensure pumping and mixing workability. While the viscosity of 15 R/20 W (3.01 Pa·s) is borderline with respect to the 3.0 Pa·s limit—especially considering standard instrumental repeatability tolerances—the 15 R/25 W blend substantially exceeds it (3.23 Pa·s). In contrast, the 15 R/15 W formulation maintains an adequate workability margin (2.22 Pa·s at 135 °C) well within the specification threshold. From a colloidal viewpoint, excess WPU induces phase demixing by expelling maltene/light components from the base asphalt, thereby increasing the effective volume fraction of the dispersed phase and causing an abrupt viscosity escalation. Therefore, to ensure acceptable workability and microstructural equilibrium, the WPU content must not exceed 15%.

### 4.3. Rheological Properties

#### 4.3.1. Temperature Sweep Test

**Figure 8:**
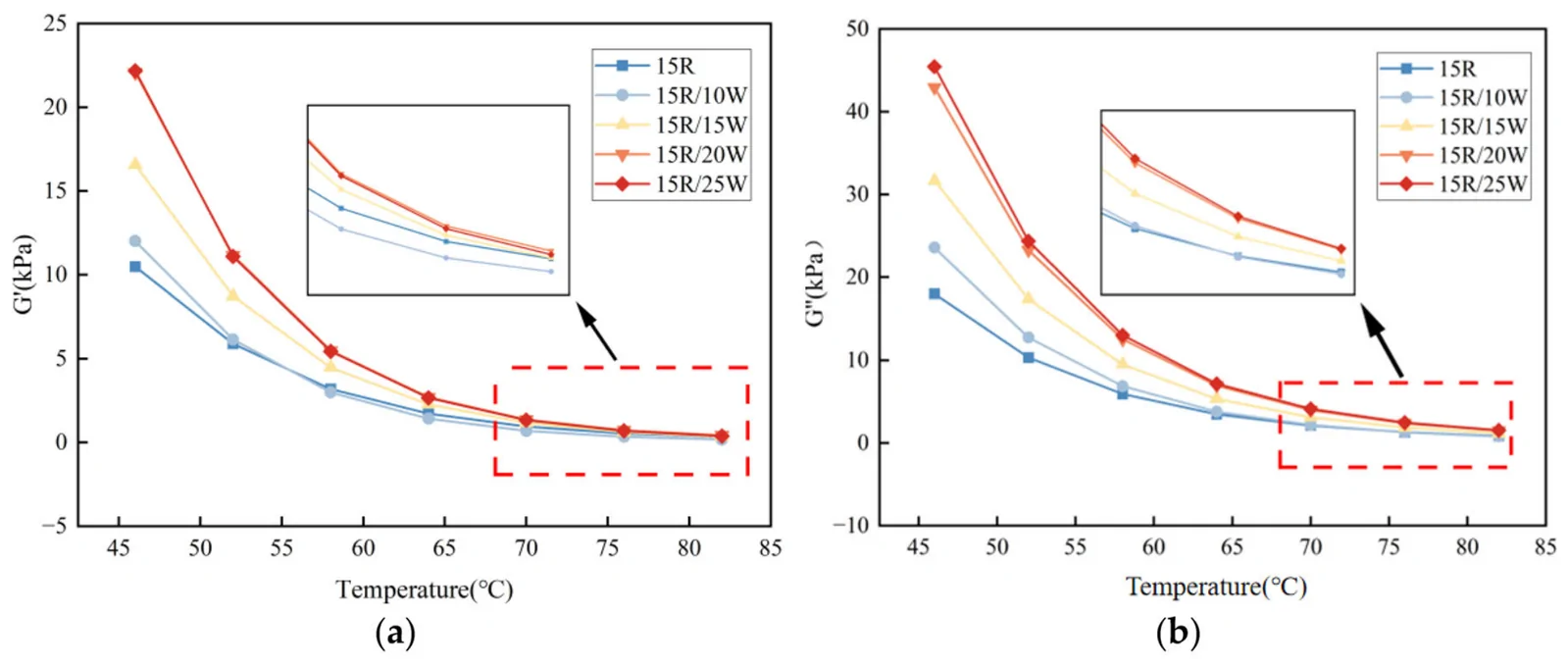
Storage modulus and loss modulus of WPU/CR composite-modified asphalt. (**a**) Storage modulus (*G*′), (**b**) loss modulus (*G*″).

As illustrated in Figure 8, blending WPU into CRMA markedly elevates both the storage modulus (*G′*) and loss modulus (*G*″). Both dynamic moduli exhibit a dosage-dependent rise, reaching peak values at 20% and 25% WPU concentrations. The negligible variance between these two highest dosages suggests a plateau effect, where surplus WPU fails to provide further modulus gains. Across the tested thermal range, *G*″ consistently surpasses *G*′ for all composite formulations, with both parameters undergoing a downward trend as temperature rises. This behavior demonstrates that WPU modification reinforces overall viscoelasticity, exerting a more substantial impact on the viscous component. Mechanistically, WPU particles disperse and partially entangle into an interlocking spatial network, bolstering viscous resistance. In parallel, the intimate physical interfacial contact and local molecular entanglement between softened WPU and swollen CR restrict polymer chain mobility, thereby bolstering the overall viscoelastic response of the composite binder.

**Figure 9:**
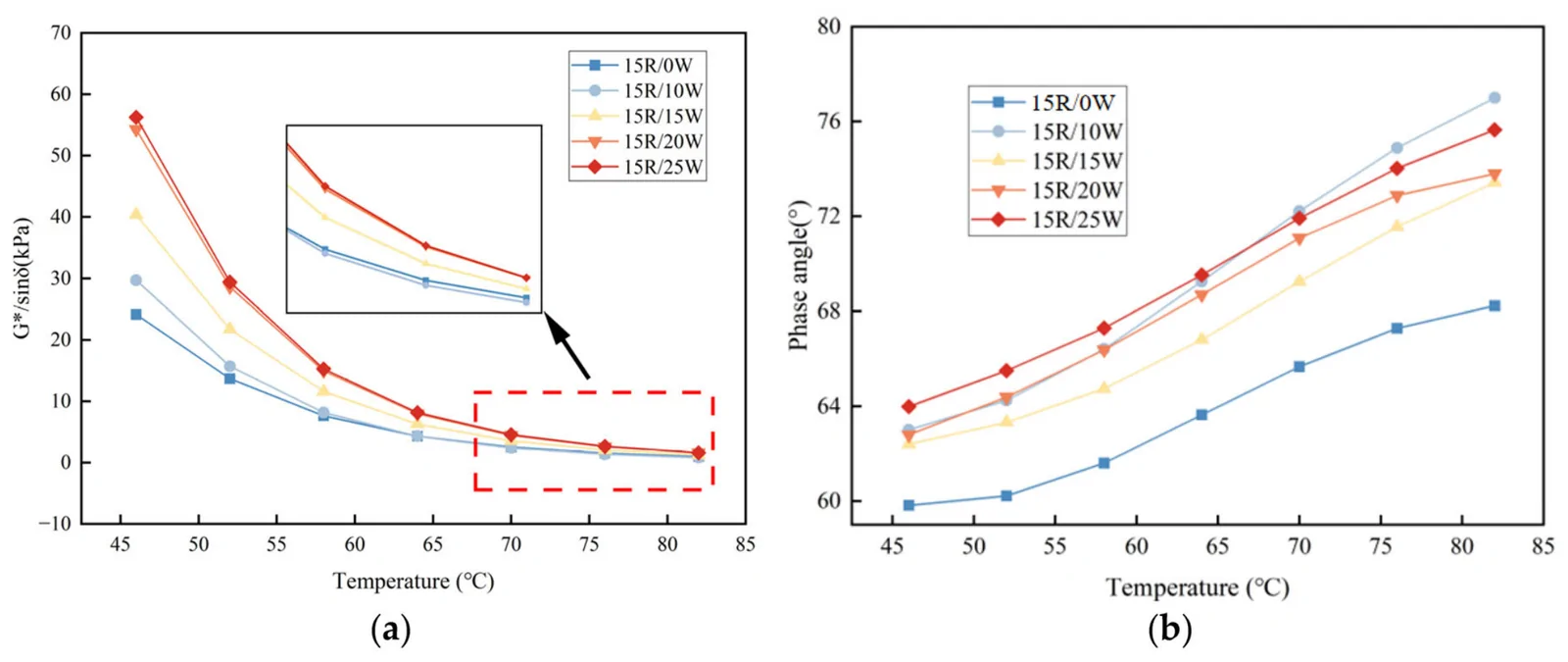
Rutting factor and phase angle of WPU/CR composite-modified asphalt. (**a**) Rutting factor (G*/*sinδ*), (**b**) phase angle (*δ*).

Figure 9 presents the temperature and dosage sensitivity of the rutting parameter (G*/*sinδ*) and phase angle (*δ*) for WPU/CR-modified binders. Elevating the WPU fraction steadily promotes G*/*sinδ*, whereas thermal escalation triggers a declining trend that eventually stabilizes into a plateau. Under the Superpave PG framework, unaged binders must maintain a G*/*sinδ* above 1.0 kPa; the blend containing 10% WPU fell short of this threshold at 82 °C [29]. Relative to control CRMA, *δ* initially climbs at lower WPU dosages, dips marginally upon further modifier incorporation, and subsequently resumes a modest upward trajectory, while exhibiting an overall increasing trend with rising temperature. These findings confirm that composite modification substantially strengthens permanent deformation resistance over single CRMA, though this reinforcing efficiency slows once the WPU dosage surpasses 20%. Although the composite binders display overall higher *δ* values than neat CRMA, modifier loading exerts only a slight fluctuating impact on *δ*. This phenomenon aligns with the stiffening effect and viscosity growth identified from dynamic modulus analysis. The co-enhancement of rutting resistance and *δ* originates from the WPU–CR spatial network, which suppresses plastic flow and markedly elevates G*. Concurrently, the elasticity provided by this skeletal network offsets thermal softening, restraining the surge in phase angle and ultimately elevating G*/*sinδ*.

#### 4.3.2. Multiple Stress Creep Recovery Test

**Figure 10:**
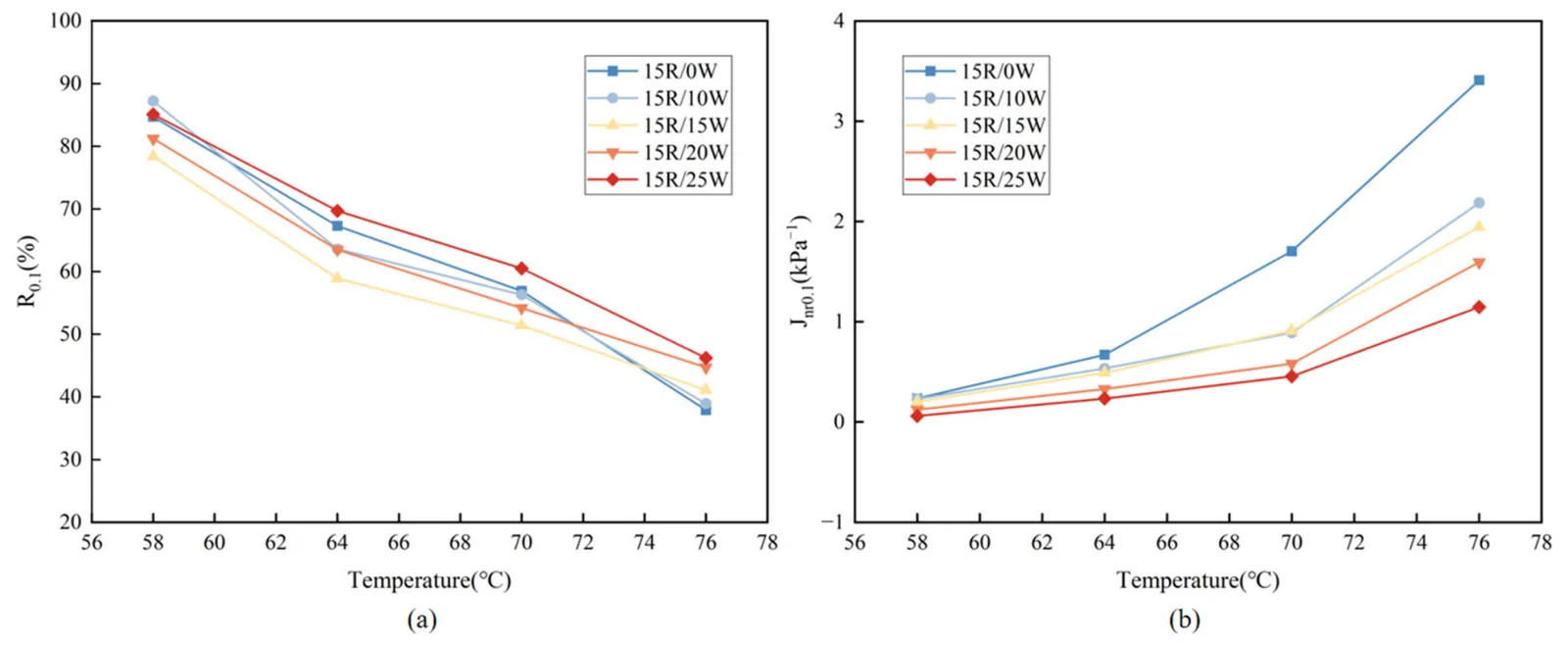
*R*_0.1_ and *J_nr_*_0.1_ of WPU/CR composite-modified asphalt. (**a**) *R*_0.1_, (**b**) *J_nr_*_0.1_.

**Figure 11:**
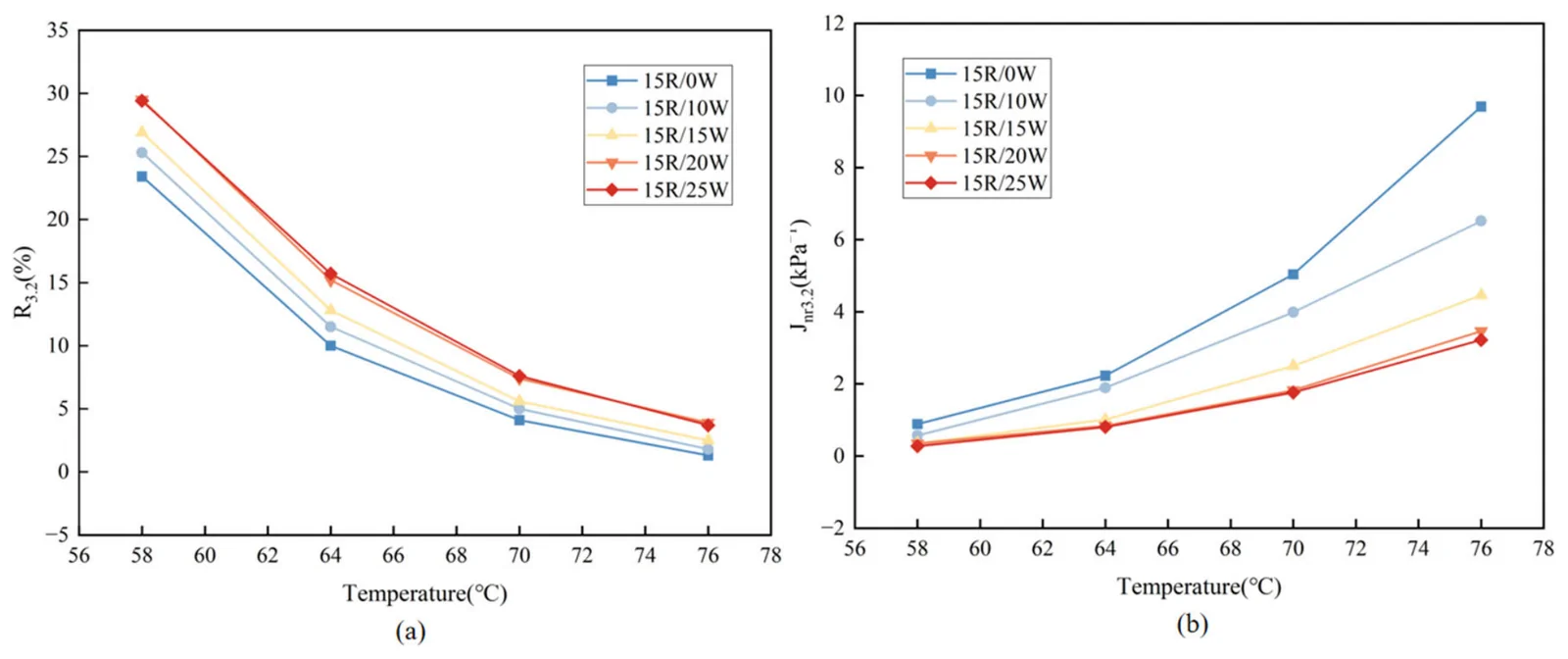
*R*_3.2_ and *J*_nr3.2_ of WPU/CR composite-modified asphalt. (**a**) *R*_3.2_, (**b**) *J_nr_*_3.2_.

Figure 10 and Figure 11 present the MSCR profiles for the WPU/CR-modified binders, showing the percent recovery (*R*) and non-recoverable compliance (Jnr) obtained at both 0.1 kPa and 3.2 kPa. At the lighter load of 0.1 kPa, the *R* value responds non-monotonically to increasing WPU concentrations, showing an initial decline before subsequently rebounding. Conversely, under the higher 3.2 kPa stress, *R* demonstrates a continuous, monotonic increase with higher WPU fractions. Furthermore, elevated testing temperatures universally compromise the elastic restorative capacity of the matrix, leading to a consistent drop in *R* across both stress regimes.

Regarding the non-recoverable compliance Jnr, an inverse pattern was detected. Increasing the WPU dosage led to a monotonic decline in Jnr across both loading conditions, although thermal elevation consistently induced higher Jnr metrics. By concurrently boosting elastic recovery and restricting permanent strain, the addition of WPU effectively bolsters the composite’s structural resilience against high-temperature rutting. Mechanistically, this enhanced strain-restoring capability stems from the intrinsic chain flexibility and elastoplastic nature of the WPU modifier, which rapidly reverses deformation once the external load is released. Ultimately, these MSCR findings strongly corroborate the preceding dynamic shear rheology observations, reaffirming the efficacy of WPU in upgrading the thermo-mechanical stability of the asphalt blends.

**Figure 12:**
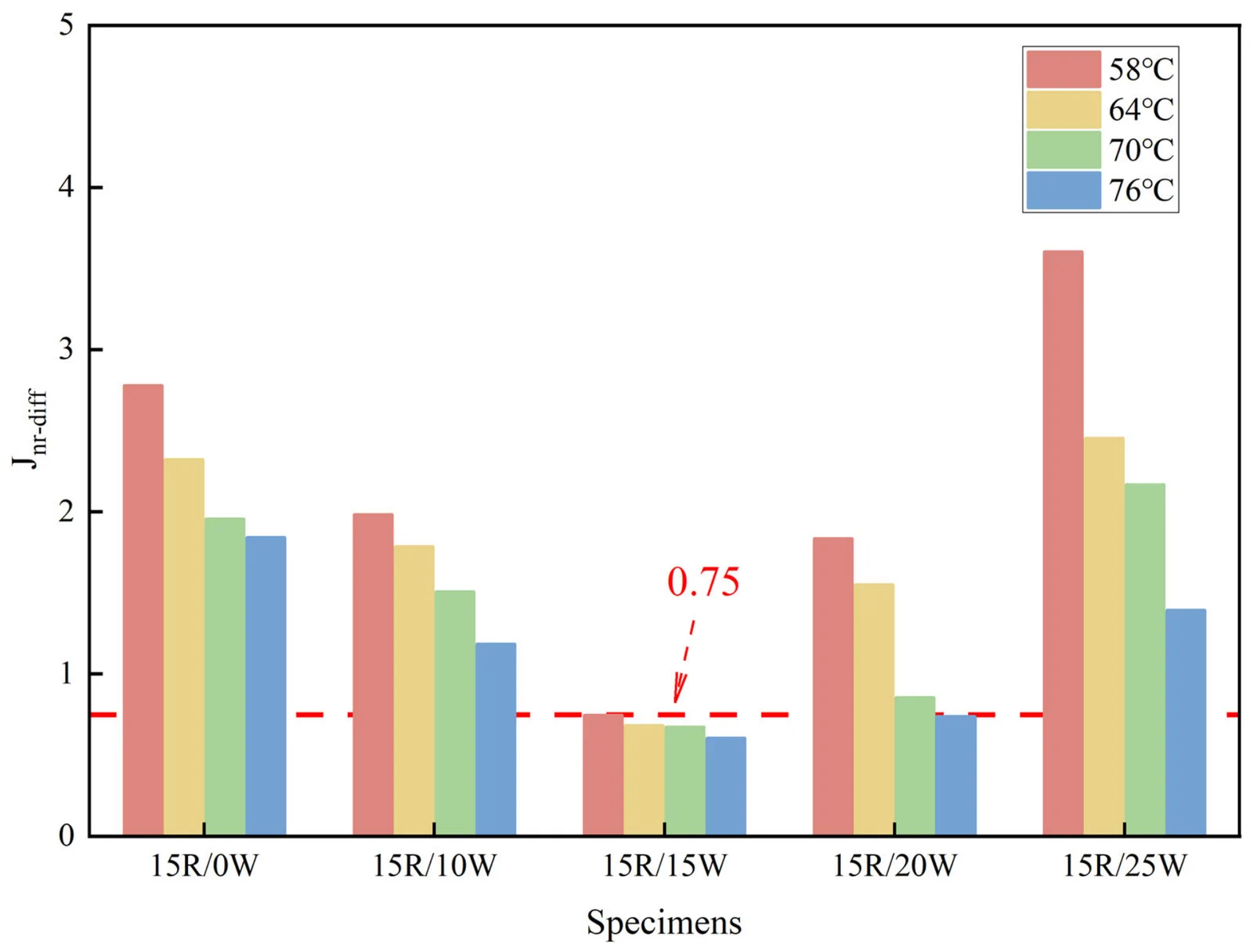
*J_nr-diff_* of WPU/CR composite-modified asphalt.

The *J_nr-diff_* of the composite-modified asphalt is governed by both modifier addition levels and environmental temperatures, as plotted in Figure 12. To prevent catastrophic shear failure under heavy and overloaded vehicle stresses, the AASHTO M332 standard [30] dictates that *J_nr-diff_* must remain ≤0.75. As the WPU fraction increases, the composite binder displays a non-monotonic *J_nr-diff_* response profile, characterized by an initial downward trend followed by a subsequent climb. Among all tested formulations, the 15 R/15 W blend exhibits superior stress insensitivity, with its *J_nr-diff_* values consistently complying with the 0.75 criterion across the complete thermal spectrum of 58 °C to 76 °C. This demonstrates that the three-dimensional physically entangled network formed by the WPU and CR particles achieves a well-balanced synergistic and compatible state, thereby significantly mitigating strain step changes under high shear stress. In contrast, 15 R/0 W and 15 R/10 W fail to establish a continuous polymer skeleton, while 15 R/20 W and 15 R/25 W induce self-aggregation and phase separation, yielding stress concentrations. Furthermore, except for 15 R/15 W, which maintains superior stability across all temperatures, *J_nr-diff_* for other binders decreases progressively with rising temperature, primarily attributed to the heightened relative deformation sensitivity between low and high stress levels at lower temperatures where the binder matrix is stiffer. Consequently, the 15 R/15 W composite-modified asphalt exhibits the most balanced deformation resistance and stability under heavy-load conditions.

### 4.4. Microstructural Characterization

#### 4.4.1. Fluorescence Microscope (FM) Test

**Figure 13:**
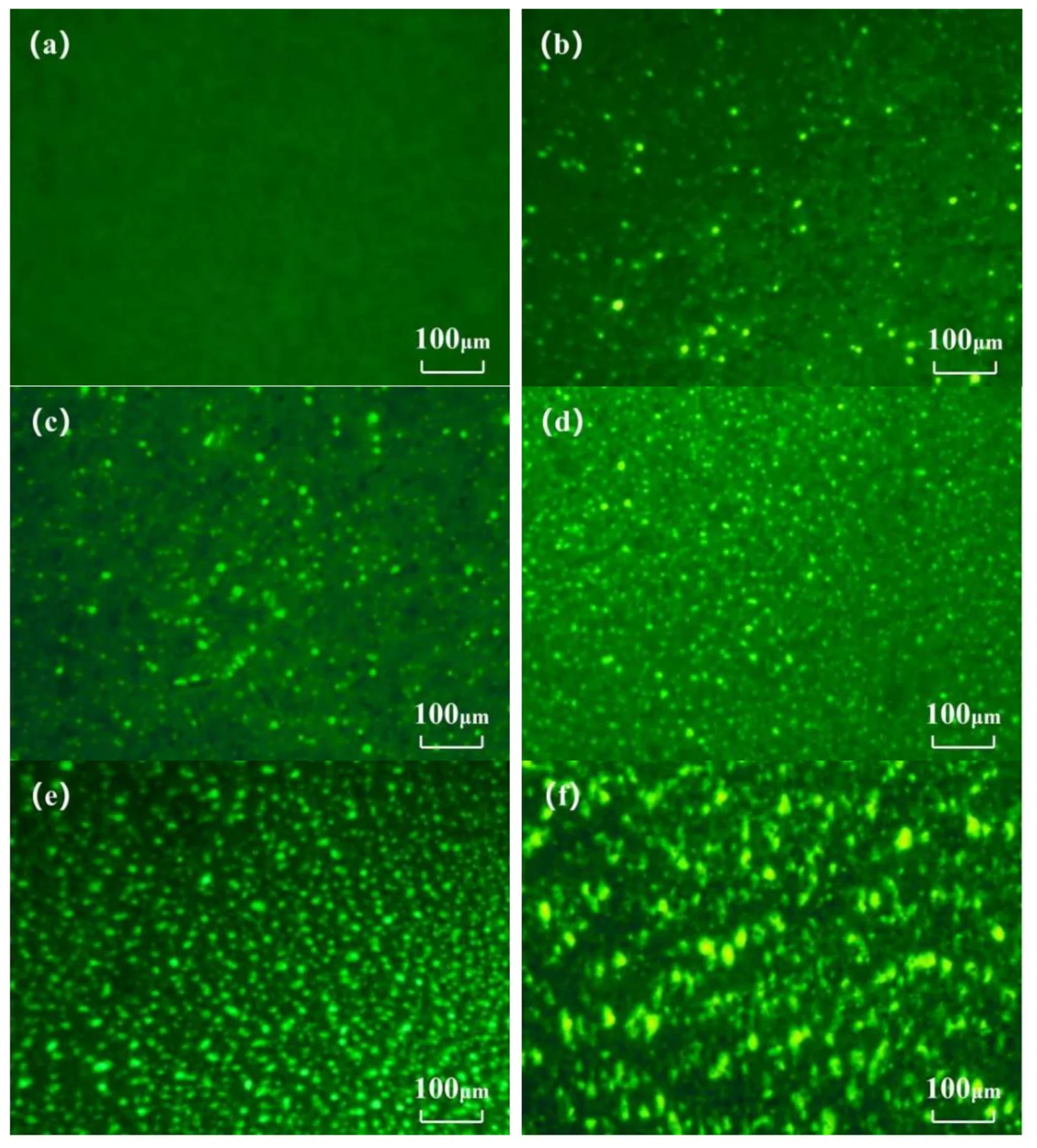
Morphology of WPU/CR composite-modified asphalt, (**a**) BA, (**b**) 15 R/0 W, (**c**) 15 R/10 W, (**d**) 15 R/15 W, (**e**) 15 R/20 W, (**f**) 15 R/25 W.

The morphological dispersion of WPU within the WPU/CR composite-modified binder is illustrated in Figure 13. Under fluorescence microscopy, the bright luminescent regions correspond to the WPU domains, while the dark green continuous background represents the asphalt matrix. The micrographs demonstrate that rather than fully dissolving into the asphalt, WPU is homogenously dispersed as discrete elastic particulates, with their spatial population density progressively multiplying at elevated modifier dosages [31]. Experimental data indicate that at WPU contents of 10% and 15%, the modifiers are finely and uniformly dispersed in the base asphalt with average particle diameters remaining <15 μm, and the fluorescent imaging area increases with higher content, demonstrating good compatibility between WPU and asphalt at these levels. However, at WPU contents of 20% and 25% (Figure 13e,f), substantial localized agglomerates and continuous polymeric clusters (>40 μm) are observed, indicating phase separation and reduced compatibility at higher particle concentrations. At moderate contents (10% and 15%), WPU domains disperse uniformly as fine elastic particulates (<15 μm) and swell by absorbing light asphalt components. However, at higher loadings (20% and 25%), the substantial swelling and severe steric crowding of high-dosage polymer particles hinder further homogeneous dispersion, triggering localized particle agglomeration and phase separation (>40 μm), which disrupts matrix continuity and elevates system viscosity [32]. This densified network structure impedes internal shear displacement, substantially enhancing the composite asphalt’s resistance to high-temperature permanent deformation. However, when the WPU content reaches 20% and 25%, severe microscopic clustering and phase demixing disrupt the continuity of the three-dimensional physical network, thereby compromising the binder’s high-temperature stability and fundamentally altering its viscosity and dynamic rheological behavior. This structural dependency aligns with recent findings that the targeted design of modifier spatial distribution within the asphalt matrix fundamentally dictates the ultimate pavement performance [33].

To establish a transparent selection rationale among the evaluated formulations, a multi-parameter decision hierarchy combining workability, stress sensitivity, and rutting resistance was employed. Although binders with 20 WPU exhibited slightly enhanced dynamic moduli, they suffered from critical drawbacks, as follows: (i) viscosities bordering or exceeding the 3.0 Pa·s workability threshold, (ii) severe non-compliance with the AASHTO M332 stress-sensitivity criterion (*J_nr-diff_* ≤ 0.75) at typical pavement temperatures, and (iii) microscopic phase separation with domain agglomerations exceeding 40 μm. By contrast, the 15 R/15 W blend strictly complied with *J_nr-diff_* ≤ 0.75 across 58–76 °C while maintaining high elastic recovery and adequate workability. Therefore, 15 R/15 W represents the best-performing formulation among the investigated samples.

#### 4.4.2. Fourier Transform Infrared Spectroscopy Test

**Figure 14:**
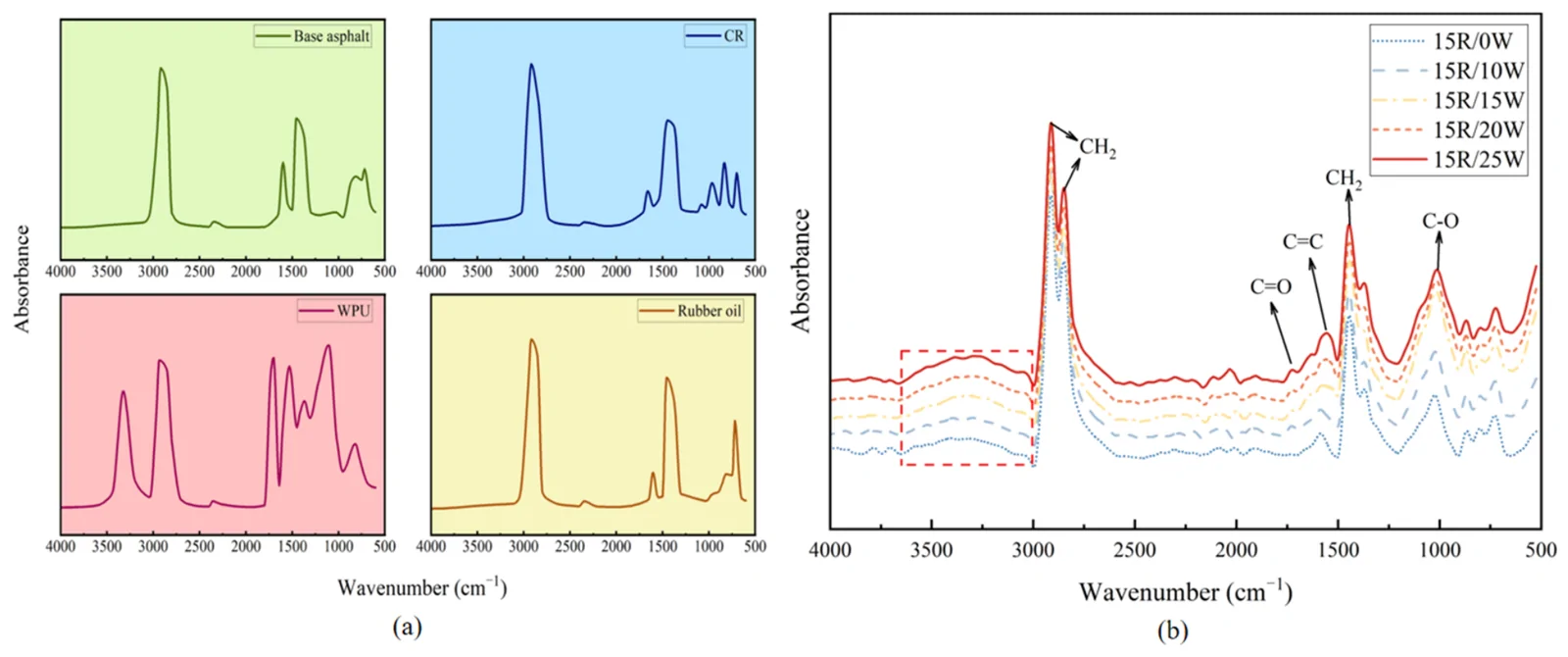
FTIR spectra of base asphalt, CR, WPU, rubber oil, and WPU/CR composite-modified asphalt with different WPU dosages. (**a**) Base asphalt, CR, WPU, and rubber oil; (**b**) WPU/CR composite-modified asphalt with different WPU dosages.

To distinguish pre-existing functional groups in the raw modifiers from potential chemical reactions during preparation, FTIR spectra of the raw constituents (base asphalt, CR, WPU, and rubber oil) and the composite-modified binders are presented in Figure 14a and Figure 14b, respectively.

As shown in Figure 14a, the raw WPU displays typical absorption bands of polyether-based thermoplastic polyurethane. The broad band around 3670–3200 cm^−1^ corresponds to –NH– and –OH stretching vibrations. The sharp peak at 1730 cm^−1^ is assigned to the carbonyl (C=O) stretching in urethane linkages (–NHCOO–), and the strong band around 1100 cm^−1^ is attributed to the ether bond (C–O–C) stretching in the polyether soft segments. In contrast, the base asphalt, CR, and rubber oil spectra are dominated by aliphatic –CH_2_– and –CH_3_ stretching vibrations at 2920 cm^−1^ and 2852 cm^−1^, as well as bending vibrations at 1455 cm^−1^ and 1375 cm^−1^, with no notable absorption in the 1730 cm^−1^ or 3200–3670 cm^−1^ regions. In addition, all four raw materials show a flat baseline between 2000 cm^−1^ and 2300 cm^−1^, confirming the absence of unreacted free isocyanate (–N=C=O, typically at ~2270 cm^−1^) in the recycled WPU.

Comparing the raw materials with the composite binders in Figure 14b, the incorporated WPU introduces its characteristic functional groups into the binder matrix without generating new absorption peaks. As the WPU dosage increases from 0% to 25%, the peaks at 1730 cm^−1^ (C=O) and 1100 cm^−1^ (C–O–C) show a progressive, monotonic increase in intensity. The broad band around 3300 cm^−1^ also strengthens; this enhancement may partly reflect the higher concentration of –NH– groups introduced by the increasing WPU dosage, and it is plausible—though not conclusively demonstrable by FTIR alone—that a portion of it arises from intermolecular hydrogen bonding between WPU and the polar fractions of the asphalt. Likewise, although no distinct new absorption peaks were detected across the entire spectrum, the absence of new peaks by itself cannot conclusively rule out the degradation of the polymer chains; it only suggests, when considered together with the TGA results in Section 2.1.3 and the morphological observations in Section 4.4.1, that high-temperature shearing at 180 °C did not trigger substantial chemical cross-linking or severe chain degradation.

Together with the rheological and morphological data, the FTIR results indicate that the modification of asphalt by WPU/CR is primarily driven by physical interactions rather than covalent cross-linking. During high-temperature shear blending, the rubber particles and softened WPU competitively absorb light fractions (saturates and aromatics) from the asphalt matrix. This swelling increases the effective volume fraction of the polymer phase while concentrating the asphaltenes in the continuous phase, leading to a stiffer binder [34]. Meanwhile, the dispersed WPU domains physically intertwine with the swollen rubber clusters to construct an interconnected polymer network, which restricts the movement of asphalt molecules and enhances high-temperature shear modulus and rutting resistance [35]. When WPU content exceeds 15%, however, severe localized agglomeration and phase separation disrupt matrix continuity, resulting in the sharp increase in rotational viscosity observed in the workability tests.

## 5. Conclusions

In this investigation, WPU/CR composite-modified binders were successfully engineered via high-shear blending. The empirical physical indices, wide-temperature viscoelasticity, and microscopic phase structure of the prepared composite binders were systematically characterized through conventional physical tests, Brookfield rotational viscometry, dynamic shear rheometer temperature sweeps, MSCR tests, and microstructural evaluations. Based on the experimental findings and mechanistic analyses, the main conclusions are summarized as follows:

(1) The incorporation of WPU successfully mitigates the thermal susceptibility of the composite binder while substantially fortifying its resistance against high-temperature rutting. However, overdosing induces unfavorable binder stiffening. As evidenced by routine empirical evaluations, selecting an appropriate WPU fraction is crucial for synergistically upgrading the overall properties of the modified asphalt system;

(2) Limited additions of WPU enhance high-temperature performance by establishing an interconnected physical polymer network within the asphalt matrix. In contrast, an overabundance of the modifier degrades the native network balance, yielding a progressively hardened composite system. Ultimately, the evidence demonstrates that carefully calibrating the WPU proportion is essential for balancing both the spatial dispersion state of the polymer phase and the mechanical characteristics of the modified binder;

(3) Incorporating an appropriate dosage of WPU substantially elevates the G* and R of the composite binder. Consequently, the high-temperature shear stability of the asphalt matrix is greatly reinforced, which effectively minimizes non-recoverable strain accumulation under cyclic shear creep loading;

(4) Morphological evaluations of the composite binder indicate that moderate WPU additions achieve uniform microscopic dispersion and favorable interfacial compatibility with both the swollen rubber and the asphalt matrix, constructing a stable 3D reinforcing network. However, dosages exceeding 15% trigger severe particle agglomeration and phase separation;

(5) Evaluating all performance metrics comprehensively, the 15 R/15 W formulation demonstrates the most favorable balance among high-temperature rutting resistance, construction workability (rotational viscosity at 135 °C), and shear stress sensitivity.

In summary, WPU/CR composite modifiers demonstrate significant potential for developing high-performance, rut-resistant asphalt binders. To bridge the gap between these laboratory findings and large-scale pavement applications, future research will focus on the modifiers’ dynamic swelling kinetics and microstructural evolution during mixing. Additionally, evaluating thermal storage stability, low-temperature performance, and comprehensive mixture performance remains essential to definitively validate their field applicability.

## Figures and Tables

**Table 1:** Properties of base asphalt.

Parameter	Results	Standards in China (JTG E20-2011)
Penetration (0.1 mm)	68	T0604
Softening point (°C)	48	T0606
Ductility (15 °C) (cm)	≥100	T0605
Dynamic viscosity at 60 °C (Pa·s)	167.5	T0620
Density (15 °C) ($g/{cm}^{3}$)	1.047	T0603
Flash point (°C)	345	T0611

**Table 2:** Properties of CR.

Property	Results	Standards
Moisture (%)	1	GB/T 19208
Ash content (%)	8	GB/T 4498
Acetone extract (%)	12	GB/T 3516
Density (g/${cm}^{3}$)	1.13~1.15	GB/T 533
Tensile strength (MPa)	15	GB/T 528
Elongation at break (%)	500	GB/T 528
Sieving rate of 80 mesh (%)	96	GB/T 19208

## Data Availability

The original contributions presented in this study are included in the article. Further inquiries can be directed to the corresponding author.
